# Blue Light Improves Stomatal Function and Dark-Induced Closure of Rose Leaves (*Rosa* x *hybrida*) Developed at High Air Humidity

**DOI:** 10.3389/fpls.2020.01036

**Published:** 2020-07-28

**Authors:** Meseret Tesema Terfa, Jorunn Elisabeth Olsen, Sissel Torre

**Affiliations:** ^1^ Department of Plant Sciences (IPV), Faculty of Biosciences, Norwegian University of Life Sciences, Aas, Norway; ^2^ School of Plant and Horticulture Science, College of Agriculture, Hawassa University, Hawassa, Ethiopia

**Keywords:** abscisic acid, blue light, darkness, relative air humidity (RH), stomata, postharvest

## Abstract

Plants developed under constant high (>85%) relative air humidity (RH) have larger stomata that are unable to close completely in response to closing stimuli. Roses (*Rosa* x *hybrida*) developed in high RH have previously been shown to have high water loss during leaf dehydration and reduced dark-induced closure resulting in a shorter postharvest life. In this study, the effect of B-light on stomatal function under high RH conditions was investigated. The ability of rose leaves developed under continuous high (90%) or moderate (60%) RH to close their stomata in response to darkness and leaf dehydration assay was studied. Moreover, the level and regulation of ABA in light and darkness in relation to B-light was measured. Our results show that increased B-light proportion improved stomatal function and dark-induced stomatal closure under high RH conditions and that was associated with increased [ABA] in general and a dynamic ABA peak during darkness. Furthermore, increased B-light during the day was associated with the presence of high β-glucosidase activity during night. This indicates that B-light is important as a signal to activate the β-glucosidase enzyme and release ABA during night. Altogether, the improved stomatal function and reduced transpiration in combination with increased [ABA] indicate that preharvest B-light plays an important role in governing stomatal functionality and ABA homeostasis under high RH and can be a useful method to improve postharvest water balance of roses.

## Introduction

In greenhouse production systems, the relative air humidity (RH) can exceed 85% in certain periods of the year (e.g. during winter) when heating costs are high and ventilation of humid air is avoided to save energy. High RH, or low aerial vapor pressure deficit (VPD), decreases the evaporative demand and transpiration rates of plants. Leaf anatomy, stomatal morphology, density, and function are reported to be modified in response to elevated RH (Torre et al., 2003; Arve et al., 2013). Plants develop larger stomata, sometimes more stomata (higher density/frequency) that are less responsive to environmental closing signals. Typically, stomata developed in high RH respond slower and do not close fully in response to darkness and/or drought resulting in plants with reduced dehydration tolerance and higher nocturnal transpiration (Torre and Fjeld, 2001; Arve, 2013; Fanourakis et al., 2013a). Excessive water loss postharvest, reduces the postharvest life and overall quality of pot roses and cut roses (Fanourakis et al., 2012; Fanourakis et al., 2013b; Carvalho et al., 2015a; Carvalho et al., 2015b). Furthermore, poor stomata control leads to early wilting during shipping and retailing and reduces marketability of ornamentals (Waterland et al., 2010a; Waterland et al., 2010b). Insight into the influence of preharvest greenhouse environment on stomatal functionality is important for enabling growth of robust, drought tolerant plants of high quality.

Different strategies to counteract the negative effects of high RH on stomatal function are reported. Some of the strategies involve technical greenhouse installations like dehumidification systems (Lim et al., 2017), selecting tolerant cultivars (Giday et al., 2013a), or modifying cultivation techniques (Fjeld et al., 1994; Fanourakis et al., 2011; Terfa et al., 2013; Giday et al., 2014; Arve et al., 2015b; Carvalho et al., 2015a; Fanourakis et al., 2016; Arve et al., 2017). Numerous studies have shown that different cultivation strategies can alleviate the negative consequences of high RH (reviewed by Fanourakis et al. (2016), such as foliar applications of the plant hormone abscisic acid (ABA) during growth (Fanourakis et al., 2011; Kim and van Iersel, 2011) and grafting on a rootstock with natural high ABA content (Giday et al., 2013a). Furthermore, modifying aerial environment such as: simultaneous changes in RH and temperature to manipulate VPD (Pettersen et al., 2007), daily changes in VPD (Arve et al., 2017); high air speed (Carvalho et al., 2015a), and changes in light quality (Terfa et al., 2012; Ahmadi et al., 2019) and irradiance (Fanourakis et al., 2019). This stimulation of stomatal functioning has been discussed to be linked to increased leaf [ABA] or changed ABA sensitivity (Aliniaeifard and van Meeteren, 2013), however it has been less pursued so far to come up with conclusive results.

The plant hormone ABA is generally known as a stress hormone, is involved in abiotic and biotic stress responses and plays an important role in the control of stomatal movement and transpiration (Wilkinson and Davies, 2002). However, ABA is also synthesized under well-watered conditions, and low concentrations of ABA affects plant metabolism and growth also under non-stressed conditions (Li et al., 2019; Yoshida et al., 2019). The endogenous level of ABA in plant tissues is dynamically regulated by the balance between its biosynthesis and inactivation (Zeevaart, 1980; Cutler and Krochko, 1999). The inactivation of free ABA involves either hydroxylation of ABA to the ABA catabolites phaseic acid (PA) and dihydrophaseic acid (DPA) or conjugation of ABA with glucose, creating ABA-glucose ester (ABA-GE) (Lim et al., 2005; Priest et al., 2006). ABA-GE is believed to be a storage form of ABA, and can be stored in the vacuoles and hydrolyzed to free ABA when required (Dietz et al., 2000; Arve, 2013). In many plant species it has been shown that ABA-GE is hydrolyzed in response to water stress, RH and darkness by *β*-glucosidase, leading to an increase in the pool of active ABA (Dietz et al., 2000; Sauter et al., 2002; Lee et al., 2006; Arve, 2013; Allen et al., 2019). In roses, conjugation of ABA with glucose, creating ABA-GE seems to be a more important inactivation pathway than hydroxylation of ABA to PA (Arve et al., 2013).

Numerous reports show that light regulates ABA biosynthesis and degradation directly or indirectly (Xiong and Zhu, 2002; Tallman, 2004; Novakova et al., 2005). Specific light qualities like B-light are also reported to regulate endogenous ABA levels during different plant developmental processes (Fellner and Sawhney, 2002; Ahmadi et al., 2019). Furthermore, the diurnal pattern of stomatal movements is affected by the diurnal alterations in metabolism of endogenous ABA, which is partly associated with the effect of light on ABA precursors such as violaxanthin (Tallman, 2004). During the day the ABA biosynthesis in guard cells is restricted by the removal of the ABA precursor, violaxanthin, through light-driven xanthophyll cycling (e.g. B light), which converts violaxanthin to zeaxanthin (Eskling et al., 1997). In light driven stomatal movements the blue (B)/Ultraviolet A (UVA) light-absorbing cryptochromes, zeaxanthin, and phototropins are the suggested receptors for B-light specific stomatal responses (Frechilla et al., 1999; Kinoshita et al., 2003; Kinoshita and Hayashi, 2011). Zeaxanthin is proposed to be a B-light-specific photoreceptor of guard cells (Talbott et al., 2003). These interconversion between xanthophyll cycles (violaxanthin and zeaxanthin) for B-light and ABA, as discussed above, shows the common denominator between the two processes. That is, both actions (B-light perception and ABA biosynthesis) could affect each other and in turn regulate stomatal movements. Hence, to understand the interactions between ABA and B-light is highly important to elucidate their implications.

In Northern greenhouse production, supplementary light is common when the natural solar radiation is low. The light is mainly supplied by gas-discharge lamp-types like high pressure sodium (HPS) lamps containing only 5% B-light in comparison to the natural solar radiation which has 15–18% B-light. It constituents of the light wavelength within the range of 565 to 700 nm, primarily yellow (565 to 590 nm) and orange (590 to 625 nm), with a peak at 589 nm (Currey and Lopez, 2013). However, during the last decade the progress in solid-state lighting, based on light-emitting diodes (LEDs) has facilitated the research on light quality responses of plants in general, and attracted much interest as a light source for assimilation lighting in greenhouses and plant factories. An important aspect of the commercial application of LEDs as supplementary lighting is how plant production can be optimized. However, attention has also been placed on other important processes like content of phytochemicals, water use efficiency (WUE), field performance and postharvest behavior (Arve et al., 2015b; Riikonen, 2016; Lanoue et al., 2017; Ahmadi et al., 2019; Leonardos et al., 2019). In spite of the progress in research on light regulation of stomatal movement, there is a lack of information on the interaction between light quality and other environmental factors. RH is economically the most difficult climate factor to control in closed production systems and the most important climate factor in determining postharvest water loss (Fanourakis et al., 2013b; Fanourakis et al., 2016). Hence, the aim of this study was to investigate the role of B-light during production in improving postharvest transpiration of roses grown under higher RH. Further, since ABA is believed to be an important signal in stress responses and stomatal movement of roses, its content and regulation in light and darkness in relation to B-light was studied.

## Material and Methods

### Plant Materials and Growing Conditions

*Rosa x hybrida*, cv. Toril plants were grown from a single node stem segment with one mature leaf as is commercially practiced. The cuttings were taken from the middle and lower position of fully developed stems with open flowers. After 2–3 weeks, the cuttings were rooted and transferred to 12 cm pots containing a standard fertilized *Sphagnum* peat media (Floralux, Nittedal, Norway). The pH and electrical conductivity (EC) level were 5.7 and 1.75, respectively, in all experiments (Superba: NPK 9-5-25+Mg+S+Mikro and calcinit, Yara, Oslo, Norway). During pre-cultivation, the plants were kept in a greenhouse compartment (glass roof and polycarbonate walls) at a temperature of 21^°^C, and average daily relative air humidity (RH) of 70% [corresponding to a water vapour deficit (vpd) of 0.74 KPa], at the Center for plant research in controlled climate at the Norwegian University of Life Sciences, Ås, Norway (N 59° 40.120’, E 10° 46.232’). Supplementary light by high pressure sodium lamps (HPS, Osram NAVT- 400W, Munich, Germany) was given 20 h every day at a photon flux density of 100 ( ± 10) μmol m^-2^ s^-1^ at 400–700 nm (measured with a Li-Cor, Model L1-185, quantum sensor, Li-Cor Inc., Lincoln, NE, USA). The pre-cultivation ended when the plants had 1–1.5 cm long shoots. Thereafter, the plants were transferred to different air humidity treatments in growth chambers.

**Figure 1:**
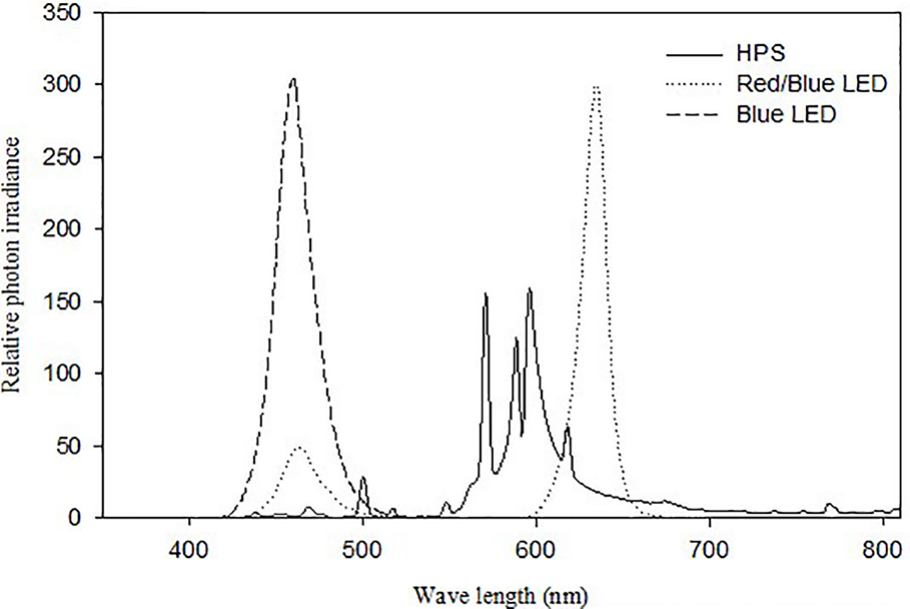
Relative spectra of the lamps used in the experiments: High pressure sodium (HPS) lamps Osram NAV T-400W (Solid lines), Light emitting diode (LED) lamps (Round LED-light 92W with 3 chains, SoLa-Co) (Dotted lines) and pure blue LED (Round LED-light 92W with three chains, SoLa-Co) (Dashed lines).

Two different experiments were carried out in controlled growth chambers and both experiments were repeated twice. The temperature set point was 20 ± 0.5°C, for all experiments during the experimental period. The RH in the growth chambers was either 60 ± 3% (moderate RH, vpd: 0.7 KPa) or 90 ± 2% (high RH, vpd: 0.23 KPa) and the CO_2_ was 400 ppm in all experiments. In *experiment I* plants were exposed to 100 µmol m^-2^ s^-1^ irradiance in a 20 h photoperiod provided either by LED lamps (round LED-light with 3 chains, Sola-co, Guangdong, China) containing 80% red (R; peak wavelength at 630 nm) and 20% blue (B) light (peak wavelength at 465 nm) or HPS lamps containing 5% B-light (HPS, Osram NAVT- 400W) in high (90%) and moderate (60%) RH (**Figure 1**). In *experiment II*, the plants were grown at high RH (90%) and subjected to 100 µmol m^-2^ s^-1^ irradiance in a 20 h photoperiod provided by a mixture of B and R LEDs with different proportions of B and R with dominant wavelength peaks at 465 and 630 nm, respectively (round LED-light with 3 chains, Sola-co, Guangdong, China). Three different spectral treatments expressed as the B-light percentage 5% B, 20% B, and 100% B were given while the remaining percentage was R. The spectra of the lamps were measured with an OceanOptics SD2000 spectrometer (**Figure 1**) (model SD2000, OceanOptics, Eerbeek, The Netherlands). The spectrometer was calibrated against a NIST-traceable calibration lamp (model LS-1-CAL, OceanOptics). Leaf temperature during growth was measured by a thermocouple thermometer (model HD 9016; Delta OHM SRL, Caselle Di Selvazzano, Italy) and was measured to be in average 1.5 °C higher under HPS compared to LED.

### Stomata Morphology Analysis

To study stomata morphology, impressions of the epidermal layer were made of fresh intact upper leaves in the middle of the day and middle of the night by Suzuki’s Universal Micro-Printing (SUMP) method using SUMP liquid and SUMP plate B (SUMP Laboratory, Tokyo, Japan) as described previously (Tanaka et al., 2005). Samples were taken interveinally close to the mid-rib on the abaxial side of the leaf from the first fully developed leaves of each plant from each air humidity and light quality treatment during both light and darkness. The SUMP imprints were observed under a light microscope (Leitz, Labolux K, Type 0.2, Wetzlar, Germany) and stomata images were obtained with a Leica camera (Leica DC200, Heerbrugg, Switzerland). Stomata morphology (length, width and area) and density were analyzed with the use of UTHSCSA ImageTool for windows version 3.00 (The University of Texas Health Science center, San Antonio, Texas, USA). The experiment was repeated twice, and twelve imprints were made from each experiment and three images were taken from each imprint for image analysis.

### Measurement of Stomata Conductance, Water Usage, and Dehydration Assay

To study the diurnal pattern of stomata conductance, measurements were done on intact fully expanded leaves for 24 h in experiment I using a CIRAS-2 Portable Photosynthesis System with PLC6 (U) Automatic Universal Leaf Cuvette (PP Systems, 2001, Amesbury, MA, USA). During all measurements, the RH and light in the leaf cuvette were the same as in the growth chamber, the CO_2_ was 400 mmol mol^-1^, the airflow 250 mmol s^-1^, and the temperature 22°C. Measurements were taken every 15 min for 24 h. The experiment was repeated twice and the measurement was taken from three plants in each repeat.

To analyze the water usage capacity, plants with intact roots were transferred from the different treatments to a test chamber with 40–50% RH, 100 µmol m^-2^ s^-1^ irradiance for 20 h photoperiod provided by Mercury lamps (Osram NAV T-400W, Munich, Germany), and a temperature of 20 ± 0.5°C. The pots were covered with plastic bags to prevent water loss through evaporation from the soil. The pots were then weighed right before dark and right after the dark period for three consecutive days. After the measurements the leaf area was determined with a leaf area meter (LI-COR, LI-3100). Leaf stomata conductance of these plants was also measured on intact fully expanded leaves using an AP4 leaf porometer (Delta-T devices LTD, Cambridge, UK). The rate of water loss (transpiration rate) per leaf area per hour was calculated as per the following equation:

$$waterusage=\frac{Changeinplantweightbeforeandafterdarkperiod}{leafarea*\mathrm{hour}}$$

Leaf dehydration assay were also done to study the stomatal response to dehydration. Detached upper leaves from eight plants grown under different RH and light quality treatments were tested in a test room with 50% RH, an irradiance of 15 µmol m^-2^ s^-1^ and 22°C. The leaves were weighed after 0, 5, 10, 15, 20, 25, 30, 40, 50, 60, 90, 120, and 180 min after detachment and their relative weight after the leaf dehydration assay were determined.

### ABA and β-Glucosidase Quantification

For analysis of ABA, *β*-glucosidase activity, fully developed leaves were sampled in the middle of the light and dark period for all treatments after 6 weeks of treatment when the plants had 1–3 open flowers. The leaf samples were immediately frozen in liquid nitrogen and stored at -80°C prior to extraction for ABA and *β*-glucosidase quantification.

Frozen leaf tissue was freeze-dried and finely ground and extracted in distilled deionized water with an extraction ratio of 1:70 (g dry weight: ml water) overnight at 5°C. [ABA] of the extract was determined using a radioimmunoassay technique as previously described (Quarrie et al., 1988).

*β*-glucosidase analysis was done based on the procedure described by Arve et al. (2013). The leaf samples were taken from the freezer (-80°C) and immediately homogenized in liquid nitrogen using a mortar and pestle. 700 mg samples were extracted for 1.5 h at 4°C in 10 ml 100 mm citrate buffer, containing 5% (w/v) PVPP, 1 mm EDTA, 14 mm mercaptoethanol, and 10% (w/v) glycerol. Samples were then centrifuged at 1,000 rpm for 4 min (Eppendorf 5810 centrifuge, Hamburg, Germany). One hundred µl of the supernatant was mixed with 1 ml 100 mm citrate buffer containing 4 mM p-nitrophenol-*β*-D-glucopyranoside (pNPG) and incubated at 37°C for 60 min (Termaks B 8054 Incubator, Bergen, Norway). The reaction was then terminated with 2 ml 1M Na_2_CO_3_ and the amount of liberated p-nitrophenol was measured spectrophotometrically at 405 nm (Helios Alpha Spectrophotometer, Thermo Scientific, Surrey, UK). The concentration was calculated using the Beer-Lambert law, Absorbance=ϵ*length*concentration, and the molar extinction coefficient for p-nitrophenol ϵ=18,300 (Dietz et al., 2000). One unit of enzyme is then defined as the amount of enzyme needed to yield 1 nmol of p-nitrophenol per hour at 37°C. The samples were collected from five plants at the middle of the light and dark periods. Each sample consisted of 5–6 young and mature leaves from a single plant.

### Statistical Analyses

Both experiments (*Experiment I and II)* were repeated twice and since the trends of the results in the experiments were similar the data are presented as combined experimental repeats unless otherwise are stated. Significant differences between means were tested for normally distributed general linear models (GLM) and Tukey’s test. Differences with p< 0.05 were considered significantly different. All statistical tests were performed in Minitab 16.1.1 (Minitab 16.1.1, windows version, State College, PA, USA).

## Results

### Effect of Light Source: LED (20% B and 80% R) Reduces the Transpiration Rate and Improves Stomatal Closure in Plants Grown Under High RH Compared to HPS

RH and light quality (LED; 20% B and HPS; 5% B) during growth significantly affected the diurnal stomata conductance (*gs*) of rose leaves (**Table 1**; *P* < 0.01). The *gs* throughout day and night was higher for leaves of rose plants under high RH-grown as compared to moderate RH (**Table 1**). However, also light source had an effect. If plants were grown under LED with a high B-light proportion (20% B) at high RH, they had 10% lower *gs* throughout the day and reached their average lowest *gs* (30 mmol^-2^ s^-1^) during the darkness as compared to plants grown under HPS with a lower B proportion (5% B), which still had higher average *gs* (48 mol^-2^ s^-1^) during dark (**Table 1**). In moderate RH however, the difference in *gs *between plants grown under LED and HPS was statistically insignificant, but still slightly lower *gs *was measured throughout the day in plants grown under LED (**Table 1**). The percent reduction in *gs *between light and darkness for LED-grown plants at high RH was higher (20%) as compared to HPS-grown plants, which was 9.6% only (**Table 1**). In addition, the calculated day: night ratio of *gs* was higher (1.7) for LED-grown plants than HPS (1.2). To understand if the change in *gs* was due to the change in stomata aperture, we analyzed the ratio of stomata size between light and darkness. In high RH the change in aperture was much higher (37.6%) in LED-grown plants than HPS where there was no significant difference (**Figure 3**). The reduced aperture in darkness in LED-plants partly explains the decrease in transpiration during darkness, entailing an improvement in stomatal function due to the prevailing environmental condition during the day.

**Table 1:** Average stomatal conductance (mmol m^-2^ s^-1^) measured by CIRAS-2 during darkness and light periods for rose leaves grown under different relative air humidity (RH) regimes (moderate RH, 60% and high RH, 90%) and light qualities provided by light emitting diodes (LED; 20% B) or high pressure sodium (HPS; 5%B) lamps.

	Moderate RH (60%)		High RH (90%)	
	LED	HPS	LED	HPS
Light	21.9^d^ ± 0.4	26.5^d^ ± 0.4	50.5^b^ ± 1.0	60.5^a^ ± 0.9
Dark	12.0^e^ ± 1.5	15.4^de^ ± 1.7	30.1^c^ ± 1.5	48.6^b^ ± 1.5
Light/Dark ratio	1.8^a^ ± 0.8	1.7^a^ ± 0.9	1.7^a^ ± 0.7	1.2^b^ ± 0.7

Data are the mean values ± SE of measurements from two experimental repeats with three replications in each and three sampling points per hour as a technical repeat (n=6). Different superscript letters indicate significant differences (P<0.05).

**Figure 2:**
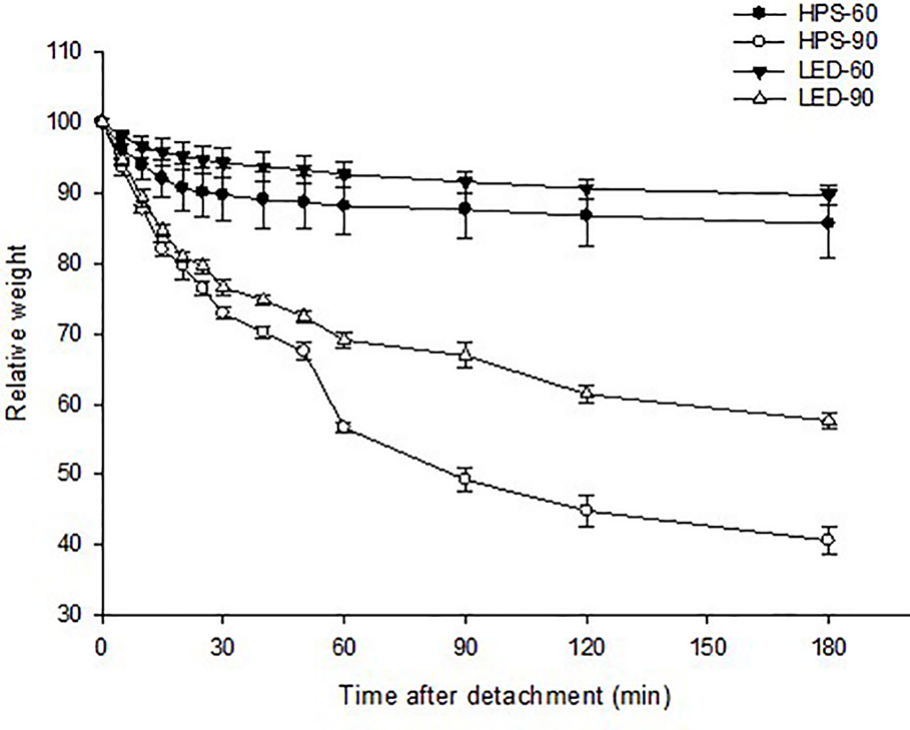
Change in relative weight (%) of detached rose leaves grown under different relative air humidity (RH) (moderate RH; 60% and high RH; 90%) and blue light proportions provided by light emitting diodes (LED; 20% B) and high pressure sodium (HPS; 5% B) lamps during 3 h of leaf dehydration assay. Data points and error bars indicate: B: leaf dehydration assay: mean ± SE from two experimental repeats with eight replications from each repeat (n=16).

Stomatal response to dehydration was tested to further analyze the degree to which the detached leaves close their stomata and retain water during a 3 h water loss test. The test showed that plants grown at moderate RH closed their stomata during the first 30 min and lost only 10% of their weight irrespective of the light quality (**Figure 2**). However, high RH-grown plants at both light qualities had lost much of their weight (40–57%), and showed a continuous transpiration throughout the testing hours as compared to moderate RH. Nevertheless, a better stomatal closure and water retaining ability was observed for the plants grown under LED. They showed about 20% less weight loss as compared to HPS-grown plants (**Figure 2**).

**Figure 3:**
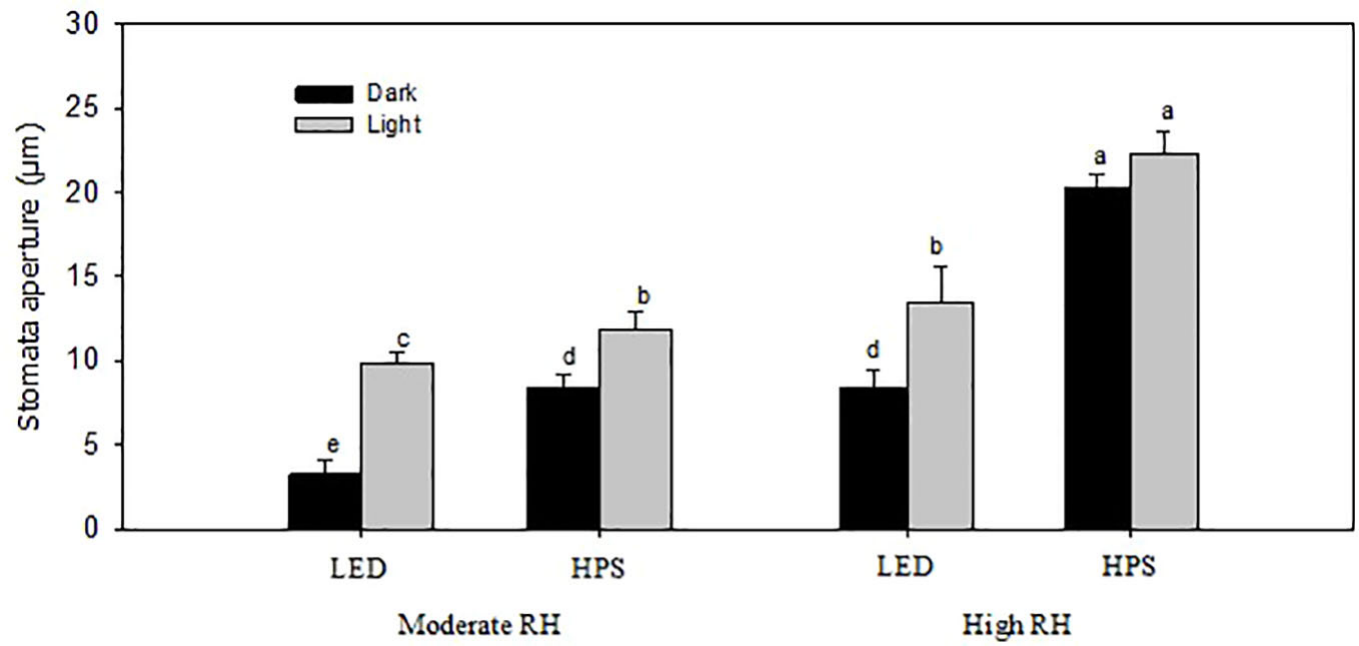
Stomatal aperture of rose leaves grown under different relative air humidity (RH) regimes (moderate RH, 60% and high RH, 90%) and light qualities provided by light emitting diodes (LED; 20% Blue) and high pressure sodium (HPS; 5% Blue) lamps. Data are the mean values ± SE from two experimental repeats with five imprint samples from each repeat and three images from each sample as technical repeat (n=1). Different letters indicate significant differences (p<0.05).

Stomata imprints of rose leaves made during the light and dark periods showed that the stomata pore length and aperture were significantly larger at high RH than moderate RH, regardless of the light source (**Figure 3** and **Table 2**; *P*<0.05). The stomata pore length and aperture of high RH-grown plants was on average 1.8 and 1.7 times higher than in moderate RH and this affected the stomata area (**Figure 3** and **Table 2**). However, for the high RH-grown plants these stomatal characteristics were much smaller for LED-grown plants than HPS (**Figure 3** and **Table 2**). The pores length and aperture were even smaller during darkness for LED-grown plants compared to HPS. The stomata pore length of high RH and HPS-grown plants was 1.2 and 1.6 times larger during light and darkness, respectively, compared to those of LED-grown plants (**Table 2**). Correspondingly, the stomata aperture of HPS-grown plants was 2.4 and 1.7 times larger than LED-grown plants during light and darkness, respectively (**Figure 3**). Consequently, this led to a larger pore area for plants grown at high RH under HPS light than LED-grown plants (**Table 2**). However, plants grown under LED had a higher number of stomata per area as compared to HPS-grown plants (**Table 2**). This indicates that, in spite of the higher number of stomata in a high B-light proportion, the stomata have a better capability to close in response to darkness.

**Table 2:** Stomatal characteristics of rose leaves grown under different relative humidity regimes (RH) (moderate RH; 60% and High RH; 90%) and blue (B) light proportions provided by light emitting diodes LED (20% B) and high pressure sodium HPS (5% B) lamps.

		Moderate RH (60%)		High RH (90%)	
		LED	HPS	LED	HPS
Pore length (µm)	Light	26.2 ± 1.0^d^	32.8 ± 1.6^c^	40.1 ± 0.4^b^	48.2 ± 0.7^a^
	Dark	21.5 ± 0.3^e^	24.4 ± 0.3^d^	34.2 ± 0.9^c^	42.9 ± 1.3^b^
Pore area (µm^2^)	Light	176.7 ± 9.2^d^	267.3 ± 8.4^c^	325.0 ± 12.7^b^	373.1 ± 18^a^
	Dark	111.3 ± 3.5^e^	166.7 ± 11.8^d^	234.5 ± 5.6^c^	370.7 ± 10.9^a^
Stomata number (µm^-2^)		75 ± 2.5 ^b^	60 ± 3.4 ^c^	85 ± 5.2 ^a^	70 ± 3.1 ^b^

Data are the mean values ± SE from two experimental repeats with five imprint samples from each repeat and three images from each sample as a technical repeat (n=10). Different letters indicate significant differences (P<0.05).

### The [ABA] and β-Glucosidase Activity Is Highly Affected by RH and Light Quality

**Figure 4:**
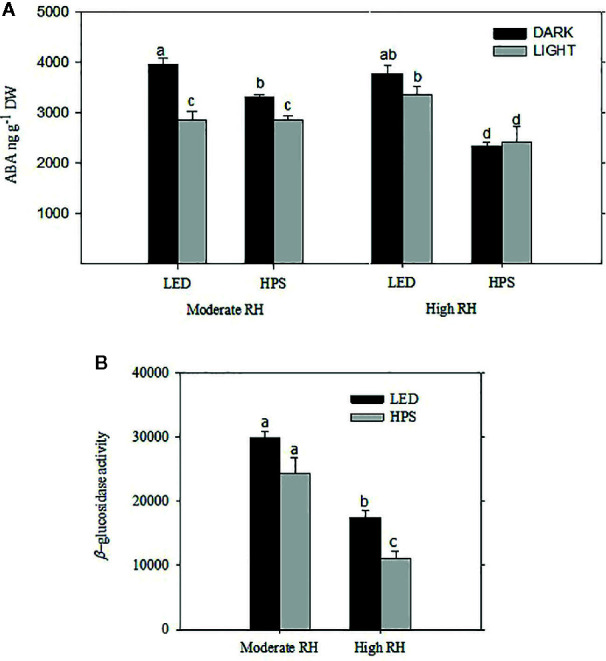
The effect of different relative air humidity (RH; Moderate RH, 60% and High RH; 90%) and blue **(B)** light proportions provided by light emitting diodes (LED; 20% B) and high pressure sodium (HPS; 5% B) lamps on the abscisic acid (ABA) content **(A)** and *β*-glucosidase activity **(B)** of freeze-dried rose leaves. For ABA quantification the samples were collected and measurements were done in the middle of the light and dark period. For *β*-glucosidase quantification, only samples from dark period were used. For both ABA and *β*-glucosidase analysis data are the mean values ± SE of 5 samples, each consisting of 5–6 leaves from each of five plants. Different letters within each figure indicate significantly different values (*P* < 0.05).

In HPS-grown plants the [ABA] was significantly higher in plants from moderate RH compared to high RH (**Figure 4A**; *P*= 0.011). In moderate RH the highest [ABA] was measured during darkness in both HPS and LED grown plants (**Figure 4A**). However, in high RH the highest [ABA] was measured in LED-grown plants, which had 40% higher total ABA level as compared to HPS-grown plants (**Figure 4A**; *P*= 0.001). The [ABA] for LED-grown plants from high RH was very much comparable to the amount of ABA measured in moderate RH (**Figure 4A**). Nevertheless, there was no significant change in ABA level between light and darkness in high RH for any of the light treatments, except a slight trend of higher ABA level during the night under LED. Hence, for plants grown under high RH and LED this slight change in ABA during light and darkness might be an important signal to induce closure in the darkness.

To further study if the increase in ABA level during darkness was related to ABA conjugation, we quantified the *β*-glucosidase activity during darkness. In our recent work with roses grown under moderate and high RH it was shown that ABA-GE, which is degraded by *β*-glucosidase, is the main catabolite playing a major role in affecting the diurnal ABA pool turnover (Arve et al., 2013). The level of *β*-glucosidase activity was significantly higher in moderate RH compared to high RH-grown plants irrespective of the light quality difference (**Figure 4B**; *P*<0.05). Furthermore, at high RH the activity of this enzyme was significantly higher under LED compared to HPS (**Figure 4B**). This could partly explain the slight trend of an increase in [ABA] in the darkness under LED.

### Effects of B-Light: Transpiration Rate, Water Usage, and Stomatal Function Is Improved as B-Light Proportion is Increased

Since HPS contains other wave lengths like yellow and orange in addition to R and B, and increases leaf temperature compared to LED (Nelson and Bugbee, 2015), another experiment (experiment II) was carried out to elucidate the sole effect of B-light on stomatal function under high RH. Plants grown under high RH were then exposed to pure B or different B-light proportions using LED lamps with distinct wavelengths of B and R-light only (**Figure 1**).

**Figure 5:**
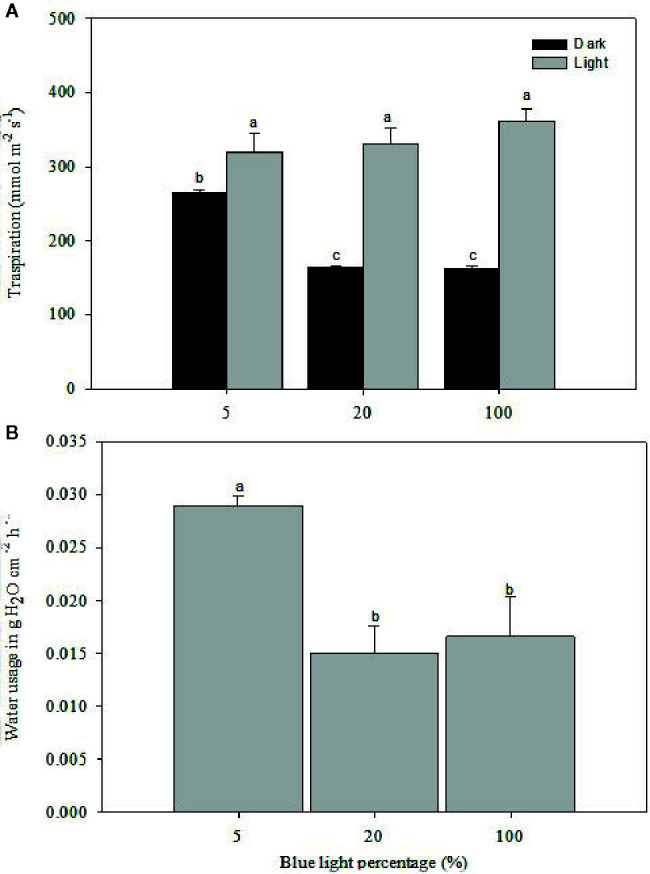
Transpiration rate during the light and dark cycle (**A**; measured by porometer) and water usage (**B**; measured gravimetrically right before and after the dark period, and data are put as a reduction in weight) of rose plants grown under high relative air humidity (RH; 90%) with different blue **(B)** light proportions provided by light emitting diodes (LED). The transpiration rate and water usage were measured for three consecutive days after plants were moved from their respective chambers to a common environment with a temperature of 20°C RH of 40% and 100 µmol m^-2^ s^-1^ irradiance for 20 h photoperiod provided by mercury lamps. The data are mean ± SE of measurements on the first fully developed leaf from ten plants. Different letters within each subfigure indicate significant differences (*P* < 0.05).

**Figure 6:**
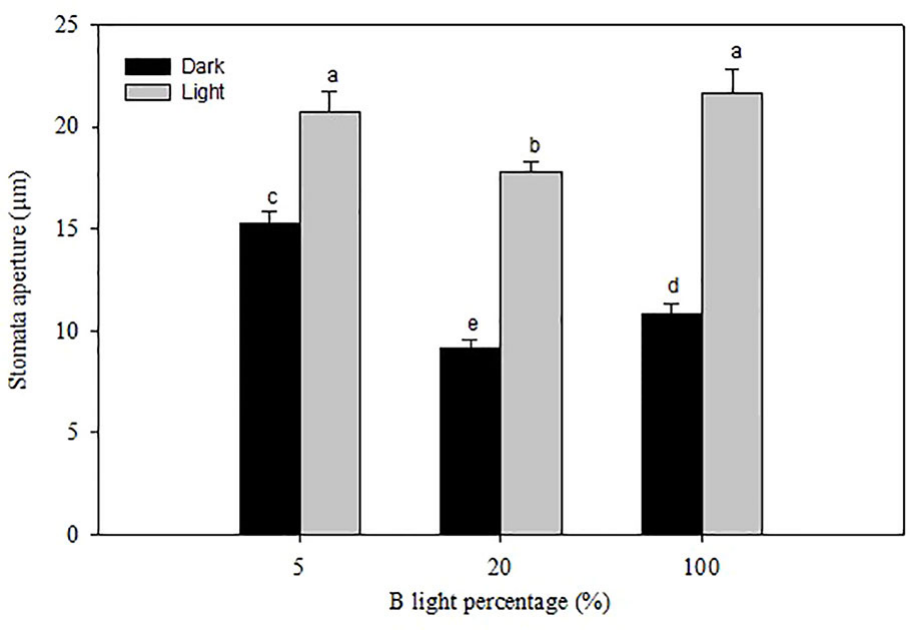
Stomatal pore aperture of rose leaves grown under and high relative air humidity (RH) with different blue **(B)** light proportions. Data are the mean values ± SE from two experimental repeats with five imprint samples from each repeat and three images from each sample as a technical repeats (n=1). Different letters indicate significant differences (*P*<0.05).

Stomatal morphology and water relations of rose leaves grown at high RH were notably affected by different B-light proportions (**Figures 5** and **6**; **Table 3**, *P*<0.01). The transpiration during the light period did not differ significantly between the different B-light proportions, only a slightly higher transpiration in the highest B proportion was observed. However, during the dark period the transpiration decreased significantly with lower *gs *(165 mmol on average) at 20% B and 100% B compared to 5% B (**Figure 5A**; *P*<0.05). The transpiration**ratio between day and night under 20% B or 100% B were 2.0 and 2.2 respectively, and this was higher than for plants grown under 5% B, which was only 1.2. This indicates a reduction in transpiration during night due to improved stomatal dark closure ability in the two highest B-light proportions.

**Table 3:** Stomatal pore characteristics of rose leaves grown under high relative air humidity (RH; 90%) and different B-light proportions.

Stomatal characteristics		B-light proportion (%)		
		5	20	100
Pore Length (µm)	ight	42.9 ± 1.4^a^	35.9 ± 1.5^c^	43.6 ± 0.6^a^
	Dark	40.7 ± 1.6^ab^	30.1 ± 0.7^d^	35.1 ± 1.1^c^
Pore Area (µm^2^)	Light	378.6 ± 21.5^a^	287.1 ± 9.4^c^	332.8 ± 12.2^b^
	Dark	265.0 ± 4.8c^d^	196.6 ± 7.2^e^	195.4 ± 8.2^e^
Stomata Number (µm^-2^)		70 ± 7.1 ^c^	85 ± 5.1^b^	113 ± 9.2^a^

Data are the mean values ± SE from two experimental repeats with five imprint samples from each repeat and three images from each sample as a technical repeat (n=1). Different letters indicate significant differences (P<0.05).

### [ABA] During Darkness Is Increased as B-Light Proportion Increases for Plants Grown Under High RH

**Figure 7:**
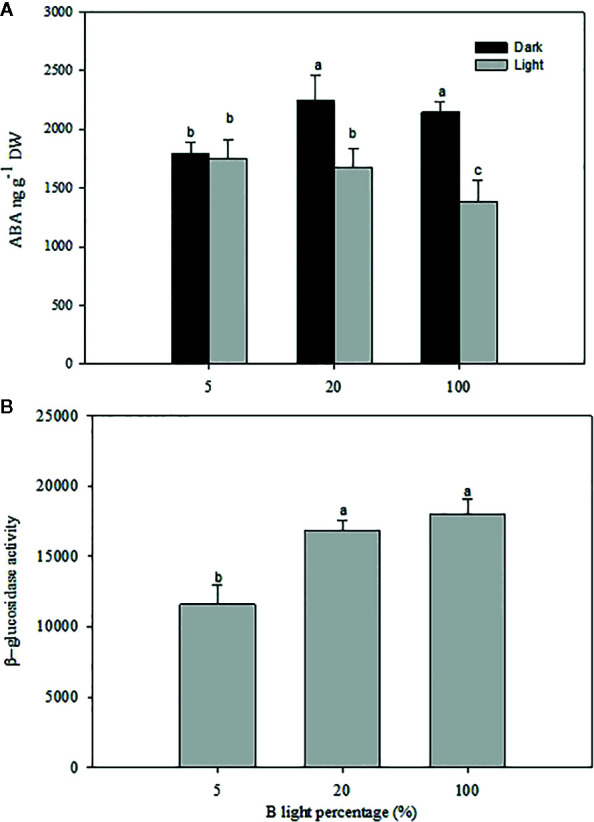
The effect of different blue **(B)** light proportions supplemented by light emitting diodes (LED) on the abscisic acid (ABA) content **(A)** and *β*-glucosidase (B) activity of freeze-dried rose leaves grown under high relative air humidity (RH; 90%). For ABA quantification the samples were collected in the middle of the light and dark period. For *β*-glucosidase quantification, only samples from dark period were used. Data are the mean values ± SE of 5 samples each consisting of 5–6 leaves from each of plants. Different letters within each figure indicate significantly different values (*P*< 0.05).

A significant difference was observed in the [ABA] in rose leaves grown at high RH under the different B-light proportions (**Figure 7**; *P*<0.05). The highest level of ABA was recorded during the dark period when plants had grown at 20 and 100% B, which was 1.2 times higher than 5% B grown plants. Whilst the lowest level was recorded during the light period for plants grown at 100% B, there was no significant difference in the amount of ABA between 5 and 20% B (*P*<0.05) during light period (**Figure 7A**). However, similar to plants grown under HPS (experiment 1, **Figure 4**), there was no significant change in the level of ABA between light and darkness for plants grown at 5% B (**Figure 7A**). The change in diurnal ABA level between light and darkness for 20 and 100% B-grown plants suggests the change in the ABA pool either by altered biosynthesis or release from conjugated ABA. To verify this, we quantified the *β-*glucosidase activity during darkness (**Figure 7B**). Hence, the quantified level of *β*-glucosidase activity was significantly higher in plants grown under 20% B or more (**Figure 7B**). *β*-glucosidase activity in 20 and 100% B plants was 1.5 and 1.6 fold higher than that of 5 % B-grown plants, respectively. This correlates with the increase in the [ABA] during darkness in plants grown under 20 and 100% B.

### Postharvest Transpiration and Responsiveness to Dry Air and Darkness as Signals for Stomatal Closure

At marketing stage (flowering), plants from the different B-light treatments were moved to a common environment with dry air (40% RH), and 100 µmol m^-2^ s^-1^ for 20 h (provided by Mercury lamps and 20 ± 0.5°C) to test the effect on preharvest light conditions on stomatal functionality and water usage. The porometer data (transpiration rate) was correlated with water usage measured gravimetrically right before and after the dark period (**Figure 5B**). Plants grown under 5% B showed 49 and 45% higher weight loss during the dark period as compared to 20 and 100% B, respectively, indicating higher transpiration rate under 5% B even during the darkness (**Figure 5B**). Thus, the data on water usage as well as transpiration suggested improvement in stomatal sensitivity during darkness in response to increased B proportions during light. To further verify this, we analyzed the stomata imprints of rose plants grown at high RH with different B proportions (**Figure 6** and **Table 3**). In all treatments the plants had open stomata during the light period and closed their stomata during darkness. During the light the stomata aperture and length were larger for plants grown under 5% B than 20% B (**Figure 6** and **Table 3**). However, no significant difference in stomata aperture or pore length during light in 100% B compared to 5% B was observed. During the night the smallest stomata aperture and length were recorded for plants grown under 20% B and 100% B (**Figure 6** and **Table 3**). The maximum percent reduction in the stomata size was recorded for plants grown under 100% B (50.1%), followed by 20% B (46.8%) and the smallest reduction was observed under 5% B (25%). This lead to smaller stomata pore area for leaves grown under 20 and 100% B as compared to leaves of 5% B-grown plants. However, the highest number of stomata per area was counted when plants were grown under more B-light (**Table 3**). Plants grown under 100% B had 1.6 and 1.3 time higher number of stomata per area than 5% B and 20% B, respectively. This might explain the slight trend of higher transpiration during day. However, the interesting point is the ability of these stomata to close during darkness so that the transpiration was reduced by more than 50% in plants grown under 20% B or more.

## Discussion

Uncontrolled water loss from plants grown at high RH leading to early wilting of leaves and flowers postharvest is common in ornamentals like cut flowers and pot plants produced in greenhouses (Mortensen, 2000; Torre and Fjeld, 2001; Fanourakis, 2011). This scenario has been suggested to have association with ABA and the diurnal pattern of stomatal response (Fanourakis, 2011; Aliniaeifard and van Meeteren, 2013; Arve, 2013; Giday et al., 2013a; Giday et al., 2014; Fanourakis et al., 2016). Preharvest factors like RH and light quality can affect the speed and degree to which stomata close or/and open after harvest. In this study we showed that a high portion of B-light (≥20%) can improve the stomatal function, a better dark-induced stomatal closure of roses grown under high RH conditions, and in turn improve postharvest water relations and product longevity.

### Blue Light Reduces the Transpiration and Improves the Stomatal Function of Roses Grown Under High RH

In the present study, the rose plants which were grown under constant high RH during leaf development clearly showed a poor stomatal closure in response to darkness and dehydration similar to what was found previously (Fanourakis et al., 2011). Nevertheless, we found that the stomatal characteristics and transpiration rates were significantly different in high RH plants exposed to LED with 20% B and 80% R as compared to under HPS (5% B) (**Figures 2** and **3**; **Tables 1** and **2**; *P*<0.05). Continuous measurements of stomata conductance during day and night and a leaf dehydration assay further confirmed that roses grown under high RH under LED had lower transpiration rate and improved dehydration tolerance (**Figure 2**; **Table 1**) as compared to plants grown with HPS lamps. Plants grown at moderate RH did not show big differences in transpiration rate and/or dehydration tolerance in response to light sources (**Figure 2**). In conclusion, the transpiration rate and the stomatal function of plants developed under high RH seems to be more dependent on the light quality than plants grown under moderate RH.

As a response to stomata-closing stimuli, the stomata of rose plants grown at high RH and 20% B and 80% R responded strongly to darkness by closing and eventually reducing transpiration compared to HPS as a light source. To further verify the role of B-light and to separate the effects of leaf temperature (Nelson and Bugbee, 2015) and the other wave lengths provided by HPS lamps, an experiment was carried out with distinct wavelengths of only B or R-light. This showed similar B-light-induced improvement in stomatal function and reduced water usage and transpiration during darkness as the light source experiment (**Figure 5**). The transpiration rate was the highest during the light period for all the treatments, but the rate was decreased during darkness by 50 and 55%, respectively, for the plants grown under 20 and 100% B as compared to those grown under 5% B (**Figure 4A**). Accordingly, the water usage recorded during the darkness, when stomata are expected to close, was lower for plants grown under 20 and 100% B (**Figure 5B**). The higher transpiration rate during the day for all treatments and a significant reduction in transpiration rate during the darkness for plants grown under a higher B-light proportion indicates B-light-induced improvement in dark closure of stomata. This suggests that stomata of plants grown under a higher B proportion have a better control of water loss when subjected to stimuli that normally induces stomatal closure. This is similar to (Blom-Zandstra et al., 1995), showing a significant reduction in water usage and *gs* during darkness for rose plants grown at 70% RH and monochromatic supplementary B-light than those grown under orange light.

In general, rose plants grown under high RH had larger stomata length and aperture, which in turn resulted in a larger stomata area as compared to moderate RH (**Figure 3**, **Table 2**). This is similar to previous studies in a number of species (Fordham et al., 2001; Torre et al., 2003; Karbulkova et al., 2008; Arve et al., 2013). Nevertheless, plants developed at high RH and higher B proportion had smaller stomata aperture and length, which in turn resulted in smaller stomata area as compared to those developed under HPS (**Figures 3** and **5**, **Tables 2** and **3**). The decreased stomata area and aperture in plants grown under 20 and 100% B partly explains the reduced transpiration rate and better water usage (Giday et al., 2013b). Furthermore, the stomata of plants developed under high RH and 20 and 100% B closed their stomata better when subjected to darkness or dehydration as compared to those grown under 5% B. However, plants grown at higher B-light proportion (20 and 100% B) had higher number of stomata as compared to 5% B (**Tables 2** and **3**). This is in agreement with the literature, including our previous work with roses (Terfa et al., 2013) where a higher B-light proportion increased the number of stomata per area as compared to HPS. Interestingly, although plants grown under higher B-light proportion had higher number of stomata which correlated with higher day time transpiration; they were able to close their stomata better during the dark period, avoiding night-time water loss (**Table 1**; **Figures 1** and **5**). The ability of the leaves of plants grown under 20% B and more to close their stomata when subjected to darkness or leaf dehydration tests shows the improved stomata ability to quickly adapt and respond to the prevailing environmental stimuli.

### Blue Light increases the Activity of β-glucosidase under High RH Conditions and affects the Diurnal [ABA]

The lack of stomatal response in plants developed under high RH is discussed to be partly due to low leaf [ABA] and/or ABA insensitivity of the leaves (Aliniaeifard and van Meeteren, 2013). In another study, we showed that roses growing under high RH regulate their ABA differently during day and night than plants grown at moderate RH (Arve et al., 2013). A higher [ABA] during night was found in rose leaves exposed to moderate RH, but not in rose leaves from high RH (Arve et al., 2013).

In the present study, we quantified higher total [ABA] in plants grown under a higher B-light proportion (20% B and more) regardless of the RH regime. However, the highest [ABA] was measured for the samples collected during the dark period. Previously, it was suggested that the increased [ABA] during darkness for plants grown under moderate RH act as a signal for stomatal closure during darkness (Tallman, 2004; Novakova et al., 2005; Arve et al., 2013). A study on the diurnal variation of ABA in *Nicotiana tabaccum* showed an increase in [ABA] after 3 h of exposure to darkness (Novakova et al., 2005). Similarly, our previous study on *Rosa x hybrida* also demonstrated an increase in [ABA] during the darkness as compared to light period in moderate RH-grown plants, but there was no change in the [ABA] for high RH-grown plants (Arve et al., 2013). In this experiment, however, we observed a significant increase in [ABA] during darkness for plants grown under high RH and higher B-light proportion (20% B and more). This showed that the light quality is influencing the diurnal ABA levels (**Figures 4** and **7**). The increase in ABA during darkness might arise either from increased ABA biosynthesis or release from ABA-conjugate. This ABA might also have been transported from the root as a long distance chemical signal (Wilkinson and Davies, 2002) or from leaf cells (as short distance signal) (Cutler and Krochko, 1999).

The diurnal pattern of stomatal movements are suggested to be affected by the altering endogenous ABA metabolism during the day (Tallman, 2004). The author also discussed that light is one of the factors affecting sources of ABA during the day. The observed lower [ABA] during light and a sharp increase during darkness in the leaves of rose plants grown under 20% B or more in this study also indicate a dynamic change of the ABA pool either by degradation, biosynthesis, or conjugation. A possible explanation could be as Tallman (2004) indicated, guard cell ABA biosynthesis restricted by the removal of the ABA precursor, violaxanthin through light-driven xanthophyll cycling which converts violaxanthin to zeaxanthin in B-light perception during the light period; since Zeaxanthin is proposed to be a B-light-specific photoreceptor of guard cells (Frechilla et al., 2002; Talbott et al., 2003). This partly explains the lower [ABA] during light in plants grown under higher proportions of B-light (20% B or more). Plausibly, during the dark period, conditions favoring endogenous guard cell ABA biosynthesis would prevail once again to maintain stomata in closed position, hence the accumulated zeaxanthin during day as a result of B- light photoreception might start to convert to violaxanthin, which would favor endogenous ABA biosynthesis by guard cells (Tallman, 2004). However, the direct relationship between B-light and ABA biosynthesis needs further studies.

In the case of high RH, it has been shown that it is mainly the inactivation of ABA which is affected rather than the biosynthesis (Okamoto et al., 2011; Arve et al., 2015a). Our previous result showed that the ABA metabolite ABA-GE is the predominant factor in changing the ABA pool during light and darkness in different RH regimes (Arve, 2013). Based on this knowledge, we assayed the *β*-glucosidase-activity to get insight if there is any inter-conversion between ABA and ABA-GE under the different light qualities. Clearly, the *β*-glucosidase assay showed a high activity of the enzyme in moderate RH compared to high RH, similarly to what was found in Arve et al. (2013). Interestingly, although plants grown at high RH generally had lower *β*-glucosidase activity, those grown under 20% B and more had a higher activity of this enzyme as compared to those grown at lower B-light proportion. The result was consistent in both experiments (exp 1and 2). This was associated with increased ABA during darkness pointing out that the increase in [ABA] during darkness might arise from ABA-GE conversion. The decreasing trend in transpiration and stomata size during darkness for plants grown under high RH and 20% B- was also linked with increased [ABA], which improved stomatal closure during darkness. Some of this increase in ABA might be root-derived ABA. However, it is also logical to assume that the stored ABA (in the form of ABA-GE) is a rapid and easy way to regulate available ABA in guard cells to quickly induce stomatal closure in response to external stimuli, whereas the bulk of transported ABA from the root might help to inhibit stomatal opening throughout the dark period/until stomata receives an over-riding signal inducing opening. This indicated an involvement of Blight in *β*-glucosidase activity which was also observed in other processes, such as phototropic responses of *Zea mays* coleoptiles (Jabeen et al., 2006).

### Blue Light Improves Dark-Induced Stomatal Closure

Night time transpiration is potentially an important factor affecting whole-plant water balance and water use efficiency (Caird et al., 2007). Attention has been given to energy consumption in greenhouses because the lack of dark-induced stomatal closure may lead to evaporative heat loss at night. Besides, when the product continues to transpire during dark storage, this may lead to aggravated water loss and a shorter postharvest life. However, from different studies it is obvious that the magnitude of water loss occurring during the night depends on the daytime growth conditions such as RH, photoperiod, light quality, and irradiance (Blom-Zandstra et al., 1995; Caird et al., 2007; Fanourakis et al., 2012; Arve et al., 2017). In the present study, we found that high RH-grown plants had higher night time transpiration compared to moderate RH plants regardless of the light quality. Nonetheless, for high RH-grown plants, dark transpiration and water usage was reduced by 50% if the plants were grown under 20% B or more (**Table 1** and **Figure 5**). This significant reduction in transpiration and water usage correlates with decreases in stomata aperture and area during darkness (**Figures 3** and **6**; **Tables 2** and **3**). Blom-Zandstra et al. (1995) also showed that higher daytime irradiance resulted in faster stomatal closure upon transition to darkness in roses, although closure was still incomplete. The spectrum of the low intensity supplementary light (25 µmol m^-2^ s^-1^) also affected gs at night, with orange supplementary light resulted in 100 and 50% higher gs as compared to control and B-light, respectively (Blom-Zandstra et al., 1995).

The increased level of [ABA] during darkness in higher B proportion might be important in the development of functional stomata. It has been shown that, the variation in the diurnal ABA is important to develop functional stomata, similar to a daily spray with ABA (Fanourakis, 2011). In our previous (Arve et al., 2013) and present studies it has been shown that the absence of a significant change in [ABA] between light and dark period in high RH-grown plants correlated with absence of stomatal closure. Hence, this absence of a dynamic ABA peak at the beginning of the dark period in high RH-grown plants might be an important closing stimulus, and vice versa for moderate RH-grown plants. In another study, we observed that detached full developed leaves produced under high RH from roses (‘Toril’), the same cultivar as used in the present study, did not respond to exogenous ABA treatment (100 µm) while the moderate RH leaves responded and closed their stomata (Carvalho et al., 2015a). This shows that the sensitivity to ABA is reduced when leaves are developed under high RH. In the study of (Giday et al., 2013a) it was found that a rose cultivar tolerant to high RH closed its stomata better during darkness than a rose cultivar less tolerant to high RH. Hence, roses grown under high RH and a higher B-light fraction behave similar as a high RH tolerant cultivar. Furthermore, the fact that B-light itself (in addition to its effect on ABA) plays a major role as a signal in stomatal movements, it can directly affect the stomatal functionality. That is, the stomata that are adapted to regular opening and closing during development under B-light would be able to close better when they are subjected to signals important for stomatal closure such as darkness, drought or ABA. Thereby, it can be suggested that both increased level of ABA and B-light in combination or individually would contribute to the improved stomatal functionality directly or indirectly.

## Conclusion

The present study shows that B-light improves the stomatal function under high RH conditions. The ABA content was higher in darkness under a light source with a high B-light proportion and this correlates with the presence of high *β*-glucosidase activity. Taken together, the improved stomatal function in combination with increased ABA level, indicate that B-light plays an important role in governing stomatal functionality and ABA homeostasis under high RH. This result has both practical and experimental implication in such a way that, lighting sources in production systems can be improved by adding extra B to reduce the water usage during growth and to minimize postharvest losses due to an improved water balance.

## Data Availability Statement

The datasets generated for this study are available on request to the corresponding author.

## Author Contributions

Conceptualization, methodology, investigation, formal analysis, and interpretation (MT, ST, JO). Validation (ST, JO). Original draft preparation (MT, ST). Review and editing (MT, ST, JO). Supervision (ST, JO). Funding acquisition and resource administration (ST).

## Conflict of Interest

The authors declare that the research was conducted in the absence of any commercial or financial relationships that could be construed as a potential conflict of interest.

## Supplementary Material

Supplementary Figure 1 Relative spectra of Mercury Lamps (Hg lamps, Osram NAV T-400W, Munich, Germany) used in the test rooms to measure post harvest water usage and analyze dark responses of Rose plants.
