# Supplementary figures and images for: Blue Light Improves Stomatal Function and Dark-Induced Closure of Rose Leaves (Rosa x hybrida) Developed at High Air Humidity
Source: Front Plant Sci. 2020 Jul 28;11:1036. doi: 10.3389/fpls.2020.01036 (PMC7399379; doi:10.3389/fpls.2020.01036)

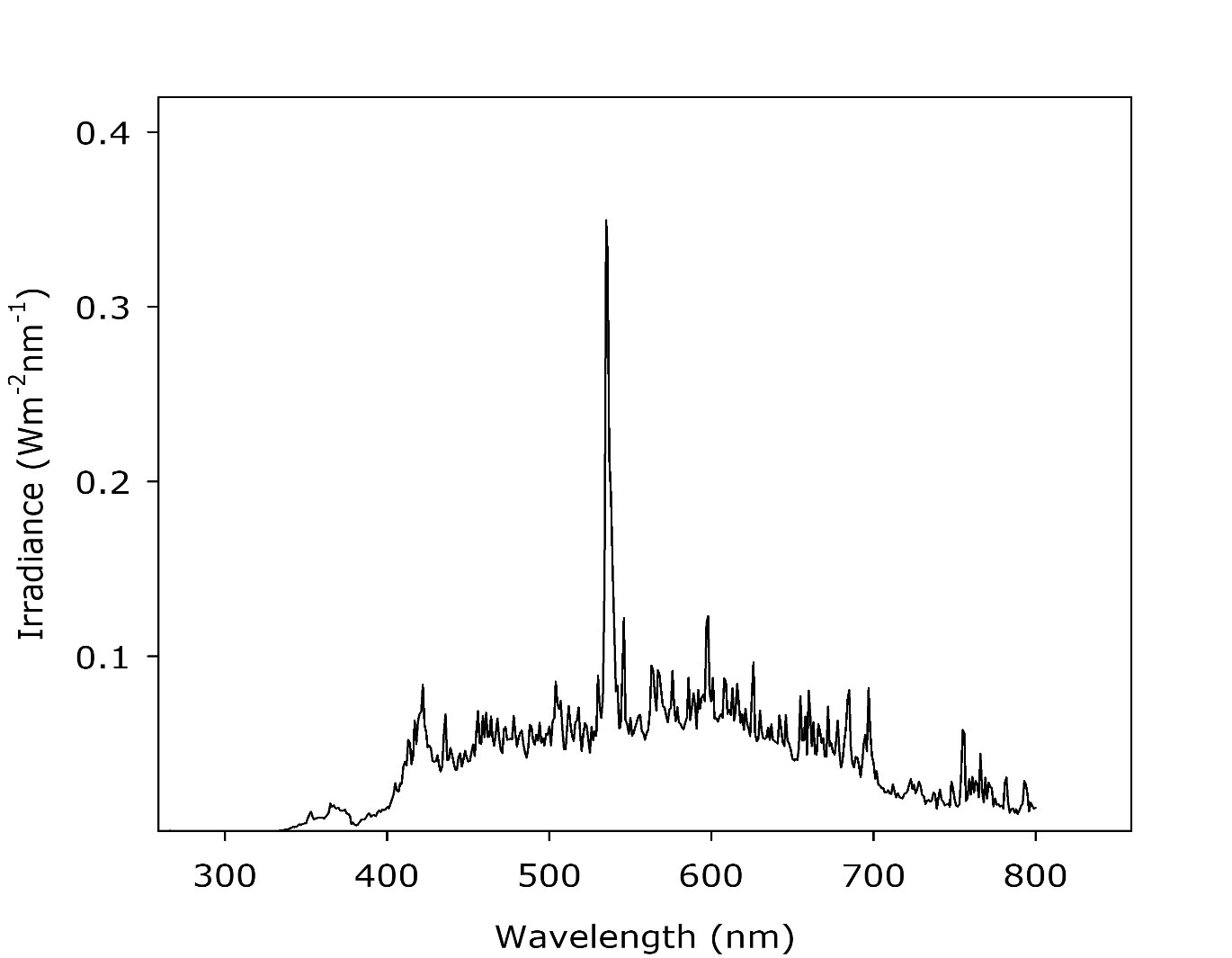

Supplement: Supplementary Figure 1 — Relative spectra of Mercury Lamps (Hg lamps, Osram NAV T-400W, Munich, Germany) used in the test rooms to measure post harvest water usage and analyze dark responses of Rose plants. [file Image_1.jpeg]
